# The effectiveness of a simplified core stabilization program (TRICCS—Trivandrum Community-based Core Stabilisation) for community-based intervention in chronic non-specific low back pain

**DOI:** 10.1186/s13018-019-1131-z

**Published:** 2019-03-22

**Authors:** Shiju Majeed A, Anish TS, Asha Sugunan, Arun MS

**Affiliations:** 1 Department of Orthopedics, Government Medical College, Trivandrum, India; 2 Department of Community Medicine, Government Medical College, Trivandrum, India

**Keywords:** Low back pain, Chronic, Exercises, Core stabilization, TRICCS protocol, Start back screening tool, ODI, Conservative

## Abstract

**Background:**

Chronic low back pain is a common public health problem all over the world. Conservative therapy is prescribed as the initial treatment strategy in chronic low back pain. The cornerstone of conservatism in back care is core muscle strengthening. However, exercises prescribed for the purpose are manifold and some are not easily done by patients in Asian countries. We developed an easy to adhere exercise protocol for core stabilization and tested its effectiveness in south Indian population.

**Methods:**

Prospective study of 73 patients with chronic low back pain (CLBP) who were subjected to Trivandrum Community-based Core Stabilisation protocol of treatment. The enrolled patients underwent initial Oswestry Disability Index (ODI) evaluation and Keele Start Back (KSB) questionnaire before starting the protocol. Back education was given, and the patient started on stratified exercise protocol. ODI assessment was done weekly. The trend in ODI changes and the factors determining them were assessed using ANOVA. The correlation of quantitative variables like age, initial ODI score, and KSB score with the rate of reduction of ODI was assessed using Pearson’s correlation. Cross-tabulations were done using the chi-square test. Parametric tests were used throughout the analysis as the quantitative study variables found to be linear. Multiple linear regression (for the quantitative outcome) and binary logistic regression (for the dichotomous outcome) were performed.

**Results:**

Mean (SD) of ODI score has reduced significantly from 43.4 (16.6) to 24.6 (17.1) over the period of 6 weeks (*p* value < 0.001). The trend in reduction of ODI scores was significantly more in KSB score less than or equal to 3 compared to KSB more than 3 even after adjusting for the general trend of decreasing ODI score over time. The reduction in ODI scores appeared to be low for advancing age (*p* = 0.468) and higher KSB scores (*p* = 0.001).

**Conclusion:**

The TRICCS protocol is effective in a community-based approach in achieving satisfactory outcomes in CLBP in a period of 6 weeks. Patients with high KSB scores may require cognitive intervention also.

## Introduction

Low back pain is a common musculoskeletal problem affecting the population both in developing and developed world. It is the major cause of work absenteeism and musculoskeletal disability [1]. Chronicity of low back pain leads to significant expenses causing strain on the health care system. Eighty percent of the population is expected to suffer from low back pain at one time or other during their life. In developed countries like the UK, the point prevalence of low back pain is estimated to be close to 50% [2, 3]. In the USA, the point prevalence ranges from 8 to 56% [4]. Various studies of low back pain epidemiology done in India give the prevalence of low back pain (LBP) between 6.2 and as high as 92% depending on the population studied [5]. In the majority of people, back pain is self-limiting. However, a significant percentage of these patients develop to have chronicity of their symptoms.

Chronic low back pain (CLBP) is defined to be back pain lasting more than 12 weeks. Chronic low back pain may be associated with true radicular symptoms in patients with canal or foraminal compromise or pseudoradicular leg pain where the pain does not fall into a particular dermatomal pattern. CLBP is generally assumed to be due to:

- Dysfunction of the motion segment of the spine (discovertebral complex and facets). This group shows varying degrees of discal degeneration in MRI as demonstrated by Pfirrmann et al. [6] or
- Nonspecific chronic low back pain which may not show significant degenerative changes in MRI.

### The aim of the study

The aim of the present study was to test the effectiveness of an exercise protocol (TRICCS protocol described later in the article) focused on core stabilization in patients with CLBP. The exercises should be easy to perform and culturally acceptable. The protocol should be easy to implement in communities with minimal resources.

The study was approved by the Institutional Research and Ethics committee. Prior written consent was obtained from the patients.

#### Inclusion criteria

Patients with CLBP (low back pain of more than 3 months duration) between the age groups 18 and 70 years who come from suburban location around Government Medical College, Trivandrum, were included in this study. They should possess at least higher secondary education. This was deemed necessary as patients had to understand the philosophy behind conservative management. Their place of residence should be within 40 min travel distance to the clinic.

#### Exclusion criteria

- Patients with other causes of back pain like fractures, tumors, high-grade spondylolisthesis, and infections.
- Patients with psychiatric illness.
- Patients who did not receive minimum higher secondary education. (The state of Kerala has a high literacy rate, and a significant proportion of its people have higher secondary education. Trivandrum is the capital city.)

All the patients were referred to our clinic from general practitioners.

During the first visit, a patient with low back pain was assessed by orthopedic spinal surgeon with radiographs and MRI. Those patients who were assigned as non specific CLBP formed part of the study.

**Fig. 1:**
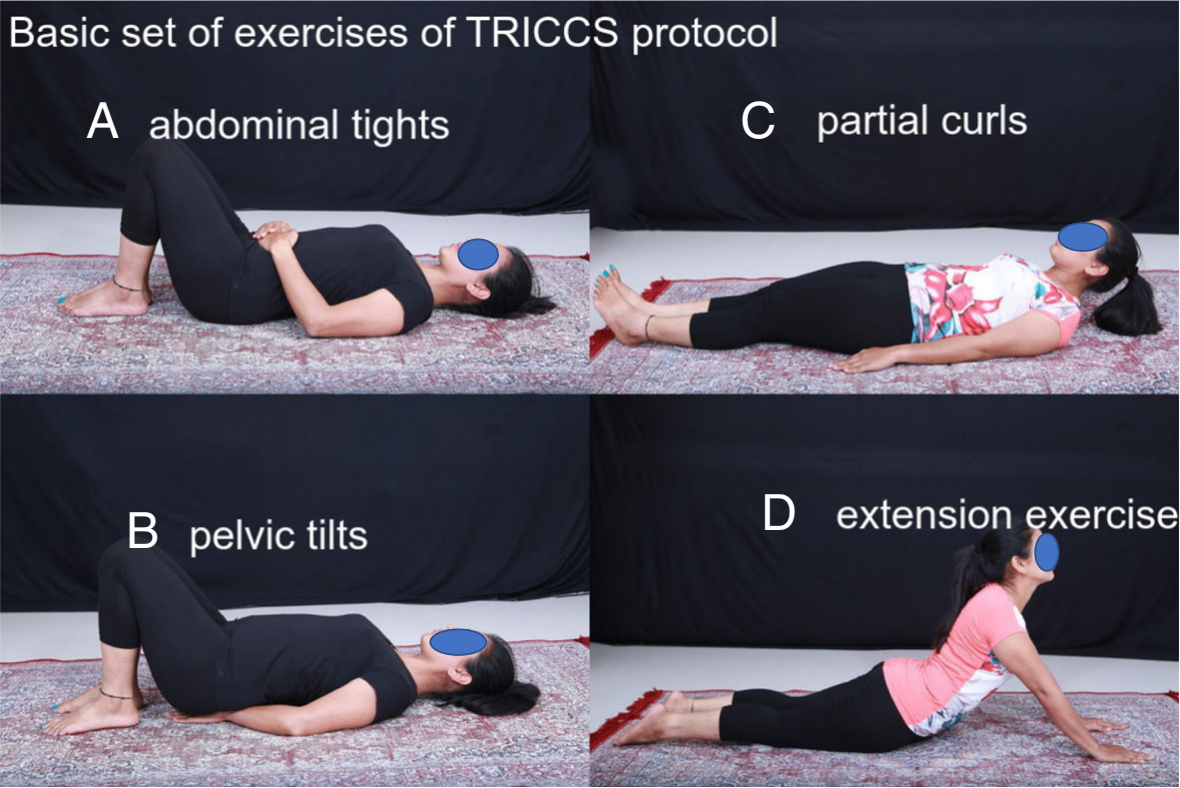
**a** Abdominal tightening—the subject is asked to do the movement which will bring the two hips together and feel for the contraction of lower abdominal muscles. **b** Pelvic tilt exercises done by keeping both hands behind buttocks and pressing on them by arching the spine. **c** Partial curl. **d** Extension (Surya Namaskar) exercises. The patient is advised to do the Surya Namaskar posture within the pain-free range

During the initial visit itself, these patients were counseled about the nature of the disease and were given a back education referring to the anatomy of the spine. Patients were encouraged to be interactive so that clear knowledge about the anatomy of the spine is acquired. They undergo the Oswestry Disability Index (ODI) assessment and Keeles Start Back (KSB) questionnaire by a nurse who is not involved in the treating group (Appendix). In the first visit, painless range of motion of spine was found out and the basic set of exercises was taught to the patient. Finding the painless range of motion of the spine is key in getting patients confidence and their cooperation while performing the exercises. The basic set of exercises was abdominal tightening, pelvic tilt, partial curl, and back extension Surya Namaskar exercises. (Fig. 1a–d). Patients were instructed to do the exercises within their painless zone. Patients were asked to progress to 20 repetitions of each exercise twice daily. This was to be done as a home program.

**Fig. 2:**
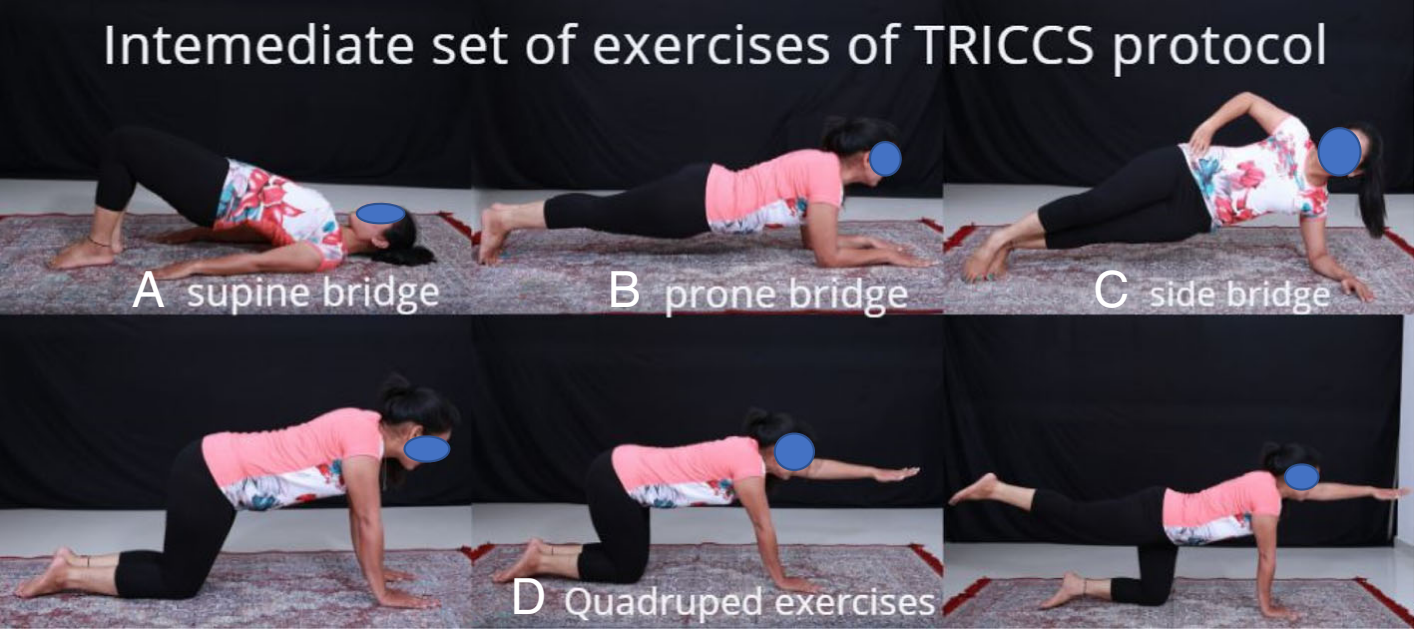
**a** Supine bridge exercises. **b** Prone bridge exercises. **c** Side bridge exercises. **d** Quadruped exercises. In the first step, the patient has to stand on all fours, then stretch one arm, then one leg, and then one arm and opposite leg in tandem

Patients are reviewed every week, and depending on the ability to perform the basic exercises, they are graduated to do the intermediate exercises. The intermediate exercises were supine bridge exercises, prone bridge exercises, quadruped, and side bridge exercises (Fig. 2a–d). Twenty repetitions twice daily was the goal.

**Fig. 3:**
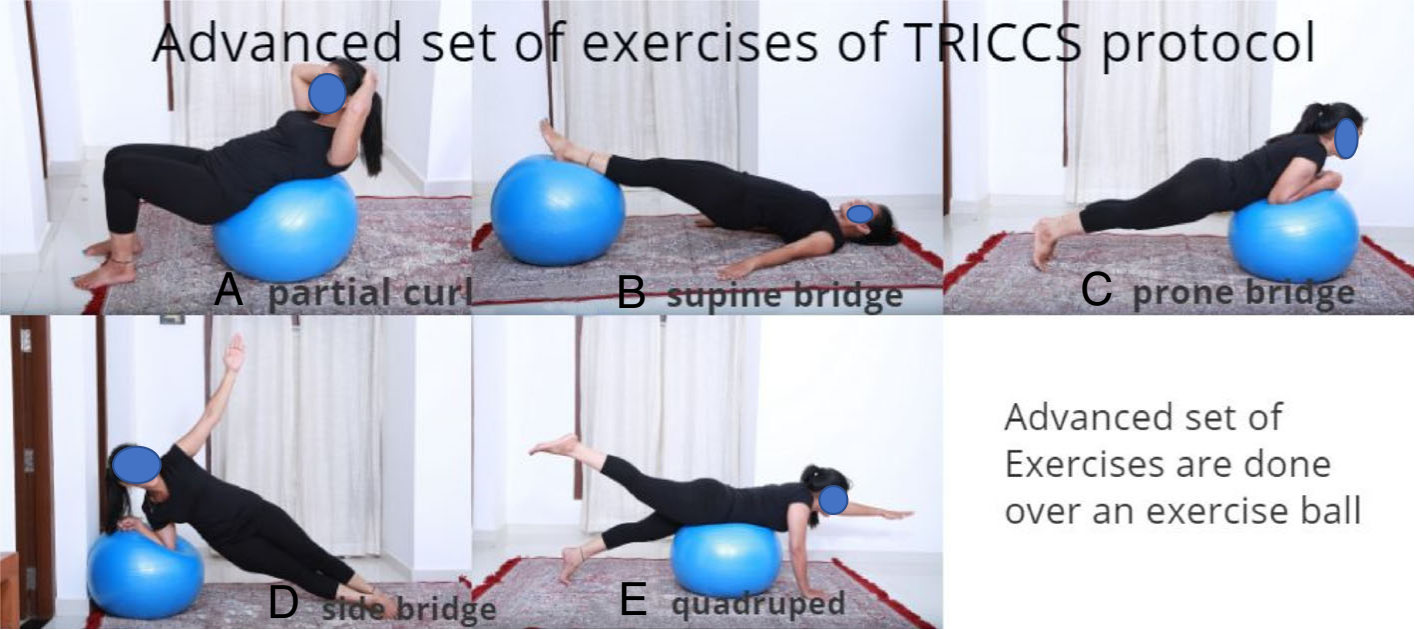
**a** Advanced core with an exercise ball: partial curl. **b** Advanced core with an exercise ball: supine bridge. **c** Advanced core with an exercise ball: prone bridge. **d** Advanced core with an exercise ball: side bridge. **e** Advanced core

Those who were able to perform the intermediate set of exercises were trained to perform the above set of exercises lying on a physio ball. These form the advanced exercises (Fig. 3a–e) and help in improving proprioceptive capability of the core muscles.

The KBS score and the weekly ODI scores were tabulated in excel sheets. Every week, patients received a phone call to remind them about the exercise that they have to do. Patients were followed up weekly in the clinic for a period of 6 weeks; thereafter, a telephonic interview was conducted at 3 months and 6 months.

## Statistical analysis

The major outcome variables of the study are the trend in ODI scores measured repeatedly, reduction in ODI score as a percentage of the initial ODI score, and the final ODI score with a score 20 or less was taken as no or minimal disability. The trend in change of ODI score over the repeated observations and the factors contributing to the difference in the trend was assessed using repeated measure ANOVA. The correlation of quantitative variables like age, initial ODI score, and KSB score with the rate of reduction of ODI was assessed using Pearson’s correlation. Independent sample *t* test was used as the statistical test to compare the mean and standard deviation of quantitative variables with a dichotomous outcome (final ODI more than 20 or not). Cross-tabulations were done using the chi-square test. Parametric tests were used throughout the analysis as the quantitative study variables found to be linear. Multiple linear regression (for the quantitative outcome) and binary logistic regression (for the dichotomous outcome) were the multivariate analyses performed.

## Results

Eighty patients were enrolled; however, 7 patients did not complete the entire duration of the study. So there were 73 patients aged 13 to 65 years with a mean (SD) age of 39.81 (12.58) years who completed the study. Forty-four (60.3%) study participants were women. Fifty (68.5%) had a KSB score of 3 or less, 21 (28.8%) had a score of 4 to 6, and 2 (2.7%) had a KSB score more than 6. The initial ODI score (ODI 1) ranged from 14 to 82 with a mean (SD) 43.40 (16.56). Final ODI value (ODI 6) ranged from 2 to 70 with a mean (SD) of 25.20 (16.39). The reduction in ODI scores from ODI 1 to ODI 6 ranged from 0 to 92.86% of the initial ODI value with a mean (SD) of 46.64 (24). The proportion of people with an ODI value less than 20 at the end of follow-up was as high as 58.90%. The number (%) of participants who had an initial value 50 or more was reduced from 26 (39.4%) to 7 (10.6%). Eight participants (30.8%) out of the 26 patients with high ODI (above 50) scores received epidural injections.

**Fig. 4:**
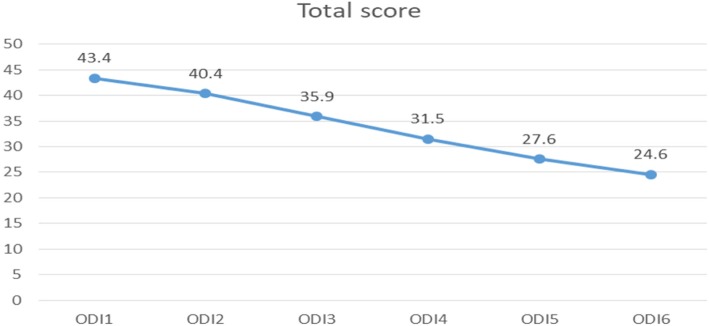
Trend in reduction of ODI scores over time (*p* < 0.001)

Figure 4 shows that the mean (SD) of ODI score has reduced significantly from 43.4 (16.6) to 24.6 (17.1) over the period of 6 weeks (*p* value < 0.001, repeated measure ANOVA). KSB score was the only significant factor associated with the difference in the trend of ODI reduction. The trend in reduction of ODI scores was significantly more in KSB less than or equal to 3 compared to KSB more than 3 even adjusting for the general trend of decreasing ODI score over time (Table 1). High scores were observed in age more than 40 years and female sex.

**Table 1:** Factors associated to trend in ODI scores

Variable	Categories	ODI 1		ODI 2		ODI3		ODI 4		ODI 5		ODI 6		*P* value
		Mean	SD	Mean	SD	Mean	SD	Mean	SD	Mean	SD	Mean	SD	
Age	Below 40 years (*n* = 42)	42.4	14.7	38.4	15.2	33.8	15.1	28.4	13.9	24.5	14.4	21.1	14.9	0.761
	40 or more (*n* = 31)	44.9	19.0	43.1	19.9	38.7	20.1	35.7	20.4	31.9	19.8	29.3	18.9	
Sex	Male (*n* = 29)	40.6	14.6	37.0	13.9	31.2	14.2	27.6	13.7	24.8	13.1	21.1	11.4	0.665
	Female (*n* = 44)	45.3	17.7	42.6	19.2	39.0	18.6	34.0	18.9	29.4	19.3	26.8	19.8	
KSB score	3 or less (*n* = 50)	39.6	14.8	35.9	14.7	30.8	13.9	26.3	13.0	22.0	11.7	18.8	10.6	0.005*
	More than 3 (*n* = 23)	52.0	17.5	50.1	19.1	46.9	19.6	42.9	19.8	39.4	20.8	37.1	21.6	

*significant *p* value <0.05

Age (correlation coefficient = − 0.236, *p* = 0.048) and KSB scores (correlation coefficient = − 0.375, *p* = 0.001) were the two factors significantly correlated to the rate of reduction of ODI scores. The reduction in ODI scores appeared to be low for advancing age and higher KSB scores. However, the initial ODI score was not significantly correlated to the rate of reduction of ODI following the treatment protocol. The mean (SD) rate of reduction for men was slightly more than that of women [48.04 (21.16) % and 44.96 (24.63) % respectively]. But the difference was not statistically significant. The epidural injection was not significantly associated with the rate of reduction of ODI in patients with initial ODI scores 50 or above. Multiple linear regression analysis revealed that KSB score is the only factor significantly correlated to the rate of reduction of ODI score adjusting for initial ODI score and age (Table 2). Achievement of an ODI scores less than or equal to 20 was considered as free of disability. Age of 40 years or less, initial ODI value (ODI 1) less than 40, and KSB score less than 3 were the three factors significantly associated with the achievement of ODI value ≤ 20. The mean (SD) value of the initial ODI for no disability and some disability were 38.89 (11.95) and 54.17 (14.33) respectively (*p* < 0.001). The mean (SD) values of KSB scores for no disability and some disability were 2.39 (1.42) and 3.62 (1.50) respectively (*p* = 0.001). Logistic regression analysis also showed that these factors were significantly associated with a disability-free outcome even adjusting for the confounding (Table 2).

**Table 2:** Independent predictors of rate of reduction of ODI scores and ODI score less than or equal to 20

Factors associated to rate of reduction of ODI scores (multiple linear regression)		
Factor	Standardized beta	*P* value
Advancing age	− 0.172	0.133
High initial ODI score (ODI 1)	+ 0.066	0.604
Lower KSB score	+ 0.401	0.002*
Factors associated to final ODI score more than 20		
Factor	Adjusted OR (95% CI)	*P* value
Age less than 40 years	33.79 (2.79–409.80)	0.006
Initial ODI score more than 40	21.39 (1.59–287.50)	0.021
KSB Score more than 3	19.70 (2.57–151.19)	0.004

*significant *p* value <0.05

## Discussion

Chronic low back pain is a major public health problem. Whether it is due to structural changes in the motion segment or due to non-specific cause, initial management is largely conservative. This involves pharmacotherapy, spinal manipulation, back education, and exercise therapy. Four types of exercise are prescribed: (a) postural exercises, (b) aerobic exercises, (c) stretching exercises, and (d) core stabilization exercises. However, most clinicians will prescribe a combination of these exercises. Exercises prescribed for the western population are not suitable to most people in suburban India. It is generally observed that patients after starting the exercise may have an exacerbation of symptoms and they drop out of the exercise therapy. Hence, it is important to train patients in the correct way of performing the exercise. The exercises should be easy to be trained and performed. They should be taught with minimum resources. The population of suburban India is not fitness freaks. The Indian Council of Medical Research (ICMR) study [7] on physical activities of people in India has shown that less than 10% of the Indian population engages in recreational physical activities. Hence, the Indian community by large is not inclined to do exercises, and it is a challenge to make people stick on to an exercise regimen. Thus, we decided to develop our community-based protocol (Trivandrum Community-based Core Stabilisation Simplifiedl—TRICCS) and test its effectiveness.

Taking the above factors into consideration, our team analyzed various core exercises and zeroed them into three sets of exercises which we grouped into basic, intermediate, and advanced.

### The concept of core and core stability

Anatomically, the core is the musculature that surrounds the lumbopelvic region and includes the abdominals anteriorly, the paraspinal and gluteals posteriorly, the pelvic floor musculature inferiorly, the hip abductors and rotators laterally, and diaphragm superiorly. All these muscles have direct or indirect attachments to the extensive thoracolumbar fascia and spinal column, which connect the upper and lower limbs). The core muscles can be divided into two groups according to their functions and attributes. The first group of muscles is composed of the deep core muscles, which are also called local stabilizing muscles. These muscles primarily include the transversus abdominis, lumbar multifidus, internal oblique muscle, and quadratus lumborum [3, 6]. The lumbar multifidus is directly connected to each lumbar vertebral segment [5], and the transversus abdominis and lumbar multifidus activate a co-contraction mechanism. The abdominal draw-in that occurs during contraction provides spine segmental stability and maintains the spine within the neutral zone [7]. In addition, these muscles provide precise motor control and are thus primarily responsible for spinal stability [6, 8]. The second group of muscles comprises the shallow core muscles, which are also known as global stabilizing muscles, including the rectus abdominis, internal and external oblique muscles, erector spinae, quadratus lumborum, and hip muscle groups [9]. These muscles are not directly attached to the spine, but connect the pelvis to the thoracic ribs or leg joints, thereby enabling additional spinal control. These muscles produce high torque to counterbalance external forces impacting the spine; thus, this group of muscles is secondarily responsible for maintaining spinal stability [6, 8, 10]. When the core muscles function normally, they can maintain segmental stability, protect the spine, and reduce stress impacting the lumbar vertebrae and intervertebral discs [11]; hence, the core muscles are also called “the natural brace” in humans [10].

Core stability is influenced by the interaction between passive, active, and neutral control systems:

- Passive system constitutes the vertebrae, intervertebral discs, zygoapophyseal joints, and ligaments.
- Active system constitutes the muscle and tendon surrounding and acting on the spinal columns.
- Neural system constitutes the nerves and CNS which directly controls the active system in providing dynamic stability [6].

#### The concept of neutral zone

The neutral zone is a region of intervertebral motion around the neutral posture where little resistance is offered by the passive spinal column. Several studies—in vitro cadaveric, in vivo animal, and mathematical simulations—have shown that the neutral zone is a parameter that correlates well with other parameters indicative of instability of the spinal system. It has been found to increase with injury, and possibly with degeneration, to decrease with muscle force increase across the spanned level, and also to decrease with instrumented spinal fixation. In most of these studies, the change in the neutral zone was found to be more sensitive than the change in the corresponding range of motion. The spinal stabilizing system adjusts so that the neutral zone remains within certain physiological thresholds to avoid clinical instability [8].

### Oswestry disability index [3]

It is a widely used tool to assess disability following back pain. The score is calculated by the addition of the values assigned for each of the 10 individual questions and is used to categorize disability as mild or no disability (0 to 20%), moderate disability (21 to 40%), severe disability (41 to 60%), incapacity (61 to 80%), and restricted to bed (81 to 100%). The minimum clinically important difference in ODI was found to be 12.8 [9]. In the present study as well, we have used ODI score and serially measured the score at weekly intervals.

The first step was to find the painless range of motion of the spine for a particular patient. Patients were encouraged to start the exercise regimen within this zone. We do not propose any new exercises that are not an already established core strengthening exercise. But we have picked few exercises that are easily doable for the Asian population and proposed a protocol which is effective in achieving improvement. As patients became accustomed to the exercise regimen, their pain-free range increased. Foundation set of exercises include abdominal tights, pelvic tilts, partial curls, and back extension. The intermediate set of exercises includes supine bridge, side bridge, prone bridge, and quadruped exercises. The advanced set includes doing the above exercises in an unstable platform like exercise ball and advanced quadruped exercises. When doing pelvic tilt exercises, patients were asked to concentrate on their deep core—transverse abdominis. They were asked to lie down in a supine position and asked to bring their hips together. This movement will contract their transverse abdominis. Patients were asked to do 20 repetitions of each exercise twice daily. Core stabilization exercises done over a physio ball has shown early and better achievement of core stability [10]. Neural adaptation with respect to body proprioception is found to occur as early as 4 weeks. The neural adaptations are the physiologic mechanisms by which torso strength and balance adaptations occur in the early phases of a conditioning training program. Hence, we have included performing on physio ball the exercises which patients have trained themselves in the initial phases of the TRICCS protocol. These constitute the advanced set of exercises. The objective of the advanced exercises on the physio ball should not be to achieve strength but to gain body control and proprioception. Neural adaptation systems will help in more efficient neural recruitment patterns resulting in coordinated motor activity and lowering of neural inhibitory reflexes [11].

It is important to use a tool which stratifies patients with chronic low back pain. CLBP has an emotional component as well, and many patients need an assessment of their psychological profile as well.

Keeles Start Back questionnaire is a simple tool to stratify patients based on their future risk of disability. Historically, anxiety, depression, fear avoidance practices, and inappropriate expectations have all been linked to chronicity of low back pain. Keele Start Back Tool helps in the stratification of CLBP patients into three groups—low risk who require no intervention other than education about back care, medium risk who will require physiotherapy, and high-risk group who will require behavioral adaptive interventions [12]. Usefulness of SBT has been validated in European ethnic groups [13, 14], but such a study is lacking in the Indian population. We tried to examine whether patients with higher Keele score performed differently in a standardized exercise regimen. If so, additional psychological interventions like cognitive behavioral therapy (CBT) would be beneficial to them, and a longer duration of exercise regimen should be thought about in the initial visit itself. This is the first study on the Indian population which used SBT as a screening tool with the aim of finding out whether high-risk group behaved differently in their outcome. We offered the same protocol to all the patients enrolled in the study and did not stratify patients based on their risk groups. As a corollary to this, we have found that patients with higher SBT values tend to have a protracted recovery. This should form part of another study where the interventions are tailored as per the risk stratification of these patients [15].

What is key to a successful TRICCS protocol is the patients’ adherence to the exercise regimen. Not only during the monitored physiotherapy schedule, but also the exercise adherence of the patient outside of the clinical setting is important. Mannion et al. [16] have shown a positive correlation between clinical outcome and exercise program adherence. Patients should be encouraged to take up the challenge of self-care, and they should be motivated to take up the responsibility of the success of their own therapy. In a community-based program, it is easier to follow up and motivate these patients. Hence, we were very strict in following up the patients and motivating them over the phone.

Our study shows that TRICCS protocol of core strengthening is able to achieve a significant reduction in back pain-related disability in a self-help model based in a community setting. Adherence to the program is also high, and significant improvement can be expected in 6 weeks. Younger age, lesser ODI score, and lower KBS score guarantee a positive result. Patients with higher ODI score and high KBS score will require longer duration, and this can be informed to the patient at the start of treatment itself.

## Conclusion

Disability due to CLBP is a growing health care task all over the world. Hospital-based settings of treating CLBP are expensive models and are not reachable to the working population in the developing world. Hence, peripheral level models have to be developed to lessen the burden of this public health problem. In developing TRICCS protocol, our attempt was to make a simple and practical management algorithm which can be easily implemented in the community using the three-tier health system which is robust in the state of Kerala.

Our results show that the TRICCS protocol is quite successful in treating chronic low back pain. What makes the protocol stand out is its simplicity which will help in community intervention for CLBP. Selected patients can be offered the service of behavioral therapists from the beginning itself so that the days of chronic pain and disability can be lessened.

## Appendix

Keele STarT Back Tool: https://www.keele.ac.uk/sbst/startbacktool/

## Abbreviations

ANOVA: Analysis of variance
CLBP: Chronic low back pain
KSB: Keele Start Back
LBP: Low back pain
ODI: Oswestry Disability Index
SBT: Start Back Tool
TRICCS: Trivandrum Community-based Core Stabilisation
